# The Expanded Invasive Weed Optimization Metaheuristic for Solving Continuous and Discrete Optimization Problems

**DOI:** 10.1155/2014/831691

**Published:** 2014-03-19

**Authors:** Henryk Josiński, Daniel Kostrzewa, Agnieszka Michalczuk, Adam Świtoński

**Affiliations:** ^1^Institute of Informatics, Silesian University of Technology, Akademicka 16, 44-100 Gliwice, Poland; ^2^Department of Industrial Informatics, Silesian University of Technology, Krasińskiego 8, 40-019 Katowice, Poland; ^3^Polish-Japanese Institute of Information Technology, Aleja Legionów 2, 41-902 Bytom, Poland

## Abstract

This paper introduces an expanded version of the Invasive Weed Optimization algorithm (exIWO) distinguished by the hybrid strategy of the search space exploration proposed by the authors. The algorithm is evaluated by solving three well-known optimization problems: minimization of numerical functions, feature selection, and the Mona Lisa TSP Challenge as one of the instances of the traveling salesman problem. The achieved results are compared with analogous outcomes produced by other optimization methods reported in the literature.

## 1. Introduction

An instance of an optimization problem is a pair (*X*, *f*), where *X* is a set of feasible solutions *x* ∈ *X* and *f* : *X* → ℝ is an evaluation function that assigns a real value to every element *x* of the search space *X*. A solution is feasible if it satisfies all constraints. The problem is to find an x* ∈ *X* for which *f*(x*) ≥ *f*(*x*) for all *x* ∈ *X* (maximization problem) or *f*(x*) ≤ *f*(*x*) for all *x* ∈ *X* (minimization problem); x* is called a globally optimal solution (or optimal solution if no confusion can occur) to the given problem instance [1].

A metaheuristic is a strategy designed to efficiently explore the search space in order to find near-optimal solutions [2]. Metaheuristics are not problem-specific and thus can be applicable to a large number of problems.

The Invasive Weed Optimization (IWO) algorithm is a metaheuristic, in which the exploration strategy of the search space is based on the transformation of a complete solution of the considered problem into another one. The authors of the original version of the algorithm from University of Tehran were inspired by observation of dynamic spreading of weeds and their quick adaptation to environmental conditions. The fundamental components of the algorithm are [3] (1) random initialization of a population of individuals, (2) reproduction dependent on the fitness of individuals, (3) random spatial dispersal of offspring, (4) competitive exclusion (selection) of individuals. In the present paper the name “IWO” will be used with reference to the original version of the metaheuristic.

Usefulness of the IWO was confirmed for both continuous and discrete optimization problems. The research was focused* inter alia* on minimization of the multimodal functions and tuning of a second-order compensator [3], antenna configurations [4], electricity market dynamics [5], a recommender system [6], and the join ordering problem for database queries [7]. The experiments described in the last mentioned paper were carried out using a modified version of the IWO which was an ancestor of the algorithm described in the present paper.

The goal of the authors is to introduce an expanded version of the IWO (“exIWO”) distinguished by the hybrid strategy of the search space exploration proposed by the authors. In addition, the IWO competitive exclusion mechanism was enriched by two variants of individuals selection, which were incorporated into the algorithm. The exIWO was evaluated by solving three optimization problems: minimization of numerical functions, feature selection, and traveling salesman problem.

The organization of the paper is as follows: Section 2 contains detailed description of the exIWO metaheuristic, Section 3 deals with the solved optimization problems and presents the outcomes of the experiments, and Section 4 summarizes the research.

## 2. Materials and Methods

Similarly to the majority of evolutionary algorithms, the idea of the exIWO can be described by the following difference equation [9]:

(1) $$\begin{array}{c} x\left[t+1\right]=s\left(v\left(x\left[t\right]\right)\right), \end{array}$$

according to which the population *x*[*t* + 1] in the time instant *t* + 1 is created on the basis of the previous population *x*[*t*] by means of operators of variation (*v*) and selection (*s*). Initial condition representing the first population *x*[0] must be defined additionally.

The simplified pseudocode mentioned in Algorithm 1 describes the exIWO algorithm by means of terminological convention consistent with the “natural” inspiration of the authors of the original IWO version. Consequently, the words “individual,” “plant,” and “weed” are treated as synonyms. It is necessary to mention that this general notation does not reveal important differences in algorithm behaviour which depend on the type of optimization problem (continuous or discrete). Details are included in the description of “dissemination” methods which constitute a set of mechanisms equivalent to the variation operator.

**Figure 1:**
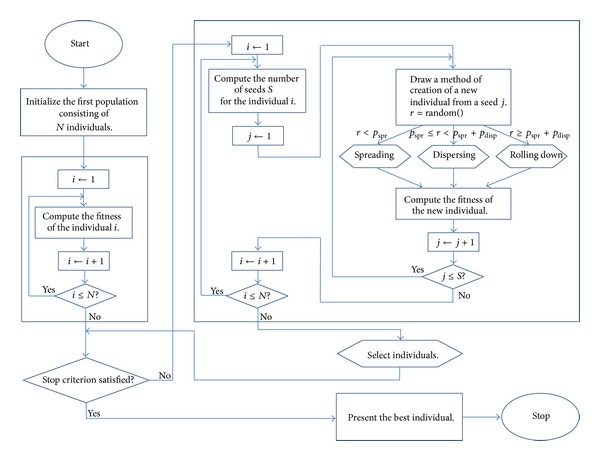
Flowchart of the exIWO.

The main intention of the authors of the present paper was to enrich the IWO's strategy of the search space exploration with components which allow for enlargement of the analyzed area as well as examination of the local extremum in the vicinity of the current point of the space. Hence, the exIWO makes use of the hybrid “dissemination of seeds” strategy, which is responsible for the “spatial dispersal,” but apart from the “dispersing” method based on the IWO's competitive exclusion it includes two additional mechanisms: “spreading” and “rolling down.” A flowchart of the exIWO is presented in Figure 1.

The optimization process starts with a random initialization of the first population. However, a greedy approach or, in general, knowledge of “good” solutions can be also considered while constructing protoplasts of individuals whose features in addition have a chance to be refined in next populations. It is worthwhile to mention that the best solution found by the exIWO cannot be of worse quality than the best one of protoplasts created in a “controlled” manner.

Stop criterion can be defined as the number of populations or as the execution time limit.

In accordance with the IWO the number of seeds *S*_ind_ produced by a single individual depends on the value of its fitness function *F*_ind_—the greater the degree of individual's adaptation, the greater its reproduction ability according to the following formula:

(2) $$\begin{array}{c} S_{\text{ind}}=S_{\min}+\left\lfloor \left(F_{\text{ind}}-F_{\min}\right)\frac{S_{\max}-S_{\min}}{F_{\max}-F_{\min}}\right\rfloor , \end{array}$$

where *S*_max⁡_, *S*_min⁡_ denote maximum and minimum admissible number of seeds generated, respectively, by the best population member (fitness *F*_max⁡_) and by the worst one (fitness *F*_min⁡_). Application of the concept of fitness and, consequently, of formula (2) is useful specially in case of maximization—the evaluation function *f* can be used directly as the fitness *F*. On the other hand, the minimized evaluation function should be rather interpreted as cost *K* which allows determining the number of seeds *S*_ind_ in the following way:

(3) $$\begin{array}{c} S_{\text{ind}}=S_{\min}+\left\lfloor \left(K_{\max}-K_{\text{ind}}\right)\frac{S_{\max}-S_{\min}}{K_{\max}-K_{\min}}\right\rfloor . \end{array}$$

According to the terminological convention the hybrid strategy of the search space exploration proposed by the authors of the present paper can be called “dissemination of seeds.” It consists of three methods randomly chosen for each seed: dispersing, spreading, and rolling down. Probability values assigned to the particular methods (*p*_spr_, *p*_disp_, *p*_roll_) form parameters of the algorithm, which should fulfill the following equation: *p*_spr_ + *p*_disp_ + *p*_roll_ = 1. The draw procedure is based on the pseudorandom number generator of the uniform distribution on the interval [0, 1). Pseudocodes for dispersing and rolling down are presented in variants designed for a discrete optimization problem (see Algorithms 2, 3, and 4).

**Figure 2:**
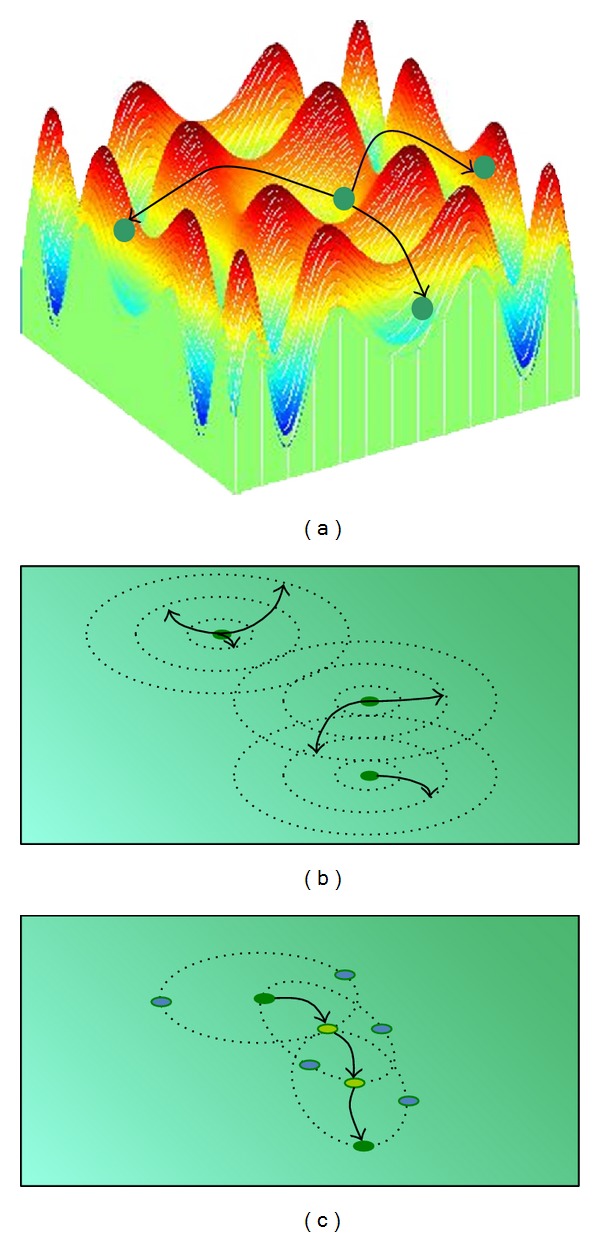
Idea of (a) spreading, (b) dispersing, (c) rolling down (*k* = 3).

The spreading (Algorithm 2) consists in random disseminating seeds over the whole of the search space (Figure 2(a)). Therefore, for a single seed this operation is equivalent to the random construction of a new individual. In other words, location of a new weed is independent of the point of the search space which represents a parent plant.

The* dispersing* (Algorithm 3) is a method based on the idea proposed in the IWO (Figure 2(b)). In case of a discrete optimization problem the degree of difference *D* between the individual *I* and his offspring can be interpreted as the distance between the parent plant and the place where the seed falls on the ground. The distance is described by normal distribution with a mean equal to 0 and a standard deviation truncated to nonnegative values. The standard deviation is decreased in each algorithm iteration (i.e., for each population) according to the following formula:

(4) $$\begin{array}{c} \sigma_{\text{iter}}={\left(\frac{\text{ite}{\text{r}}_{\max}-\text{iter}}{\text{ite}{\text{r}}_{\max}}\right)}^{m}\left(\sigma_{\text{init}}-\sigma_{\text{fin}}\right)+\sigma_{\text{fin}}, \end{array}$$

where iter denotes the current iteration (iter ∈ [1, iter_max⁡_]). Consequently, the distance is gradually reduced. The number of iterations (iter_max⁡_) can be either used as one of the algorithm parameters with the purpose of determination of the stop criterion or can be estimated based on the stop criterion defined as the execution time limit. The symbols *σ*_init_, *σ*_fin_ represent, respectively, initial and final values of the standard deviation, whereas *m* is a nonlinear modulation factor. Taking into account that the distance between plants can be interpreted as the number of transformations of the parent individual, value computed by the normal distribution generator is rounded to the nearest integer value. A transformation of an individual is a simple operation transmuting him into a different individual. Mutation is an example of the transformation. Specific character of the applied transformations depends very strongly on the optimization problem.

The rolling down (Algorithm 4) is based on the examination of the neighbourhood of the parent individual and can be interpreted as movement of a seed towards a “better” location with respect to the fitness function. The term “neighbours” stands for individuals located at the distance equal to one (transformation) from the current plant. The best adapted individual is chosen from among the determined number of neighbours, whereupon its neighbourhood is analyzed in search of the next best adapted individual. This procedure is repeated *k* − 1 times (*k* is a parameter of the method) giving the opportunity to select the best adapted individual found in the last iteration as a new one (Figure 2(c)). The parameter *k* represents also the number of neighbours taken into consideration in a single iteration of the rolling down. Thus, the method enables exploration of the vicinity of the parent individual's location in the search space.

In continuous optimization problems the distance between the parent plant and the place where the seed falls on the ground, computed by the normal distribution generator constitutes the basis for both the dispersing and the rolling down. Construction of a new individual starts with the random generation of values assigned to particular elements of the structure representing the individual (e.g., arguments of *n*-dimensional function). These values determine the direction of the seed's “flight.” Because the seed has to fall on the ground at the determined distance from the parent plant, the values of particular elements are scaled so that this condition is fulfilled. The new individual created this way by the dispersing can be also used in the rolling down procedure as one of the neighbours.

Candidates for next population are selected in a deterministic manner according to one of the following methods: global, offspring-based, and family-based. Set of candidates for the* global *selection consists of all *μ* parent plants and all *λ* their newly created descendants. This approach, which was a basis for the IWO competitive exclusion mechanism, is commonly denoted in the literature of evolutionary algorithms as (*μ* + *λ*) [9]. By contrast, the offspring-based selection, described as (*μ*, *λ*), *λ* ≥ *μ*, is limited solely to the set of *λ* descendants and thus should decrease the risk of stagnation at nonoptimal points in the search space [9]. If the best individual so far was grown in the current population, then despite the fact that it cannot be retained in the next population it will be stored with an eye to the final optimization result. According to the rules of the family-based selection, based on the idea of the originators of the* inver-over* operator [10], each plant from the first population is a protoplast of a separate family. A family consists of a parent weed and its direct descendants. Only the best individual of each family survives and becomes member of the next population. The family-based selection can be interpreted as a specific variant of the global concept which gives a chance for the preservation of characteristic features of the family. This assumption implicates a marginal importance or even absence of the random oriented spreading and also stimulates to create a well-considered method for initialization of the first population. For all the aforementioned selection methods cardinality of a population remains constant in all algorithm iterations.

Essential differences between IWO and exIWO were collected in Table 1. Introduced modifications were tested on the basis of some important optimization problems.

## 3. Results and Discussion

The goal of the research was to adapt the exIWO metaheuristic for solving three optimization problems: minimization of numerical functions, feature selection, and the Mona Lisa TSP Challenge, to conduct experiments and to compare their results with analogous outcomes produced by other optimization methods reported in the literature. Feature selection evaluated on the basis of classification accuracy belongs to maximization problems, whereas the Mona Lisa TSP Challenge requires minimization of evaluation function.

The workstation used for experiments is described by the following parameters: 2 × Intel Xeon E5620 2.40 GHz RAM 16 GB MS Windows Server 2008 R2 Datacenter 64-bit SP1.

### 3.1. Minimization of Numerical Functions

The optimized multidimensional functions: sphere, Griewank, Rastrigin, and Rosenbrock, are frequently used as benchmarks which allow comparing the experimental results with those produced by other algorithms. The minimum values found by the exIWO were confronted with the outcomes generated by the IWO and by the genetic algorithm (GA) as well as with the results of the standard Particle Swarm Optimization (SPSO) reported in the literature. For comparative purposes initial scope of the search space for the exIWO was determined each time by the conditions proposed by the authors of the referenced articles.

**Figure 3:**
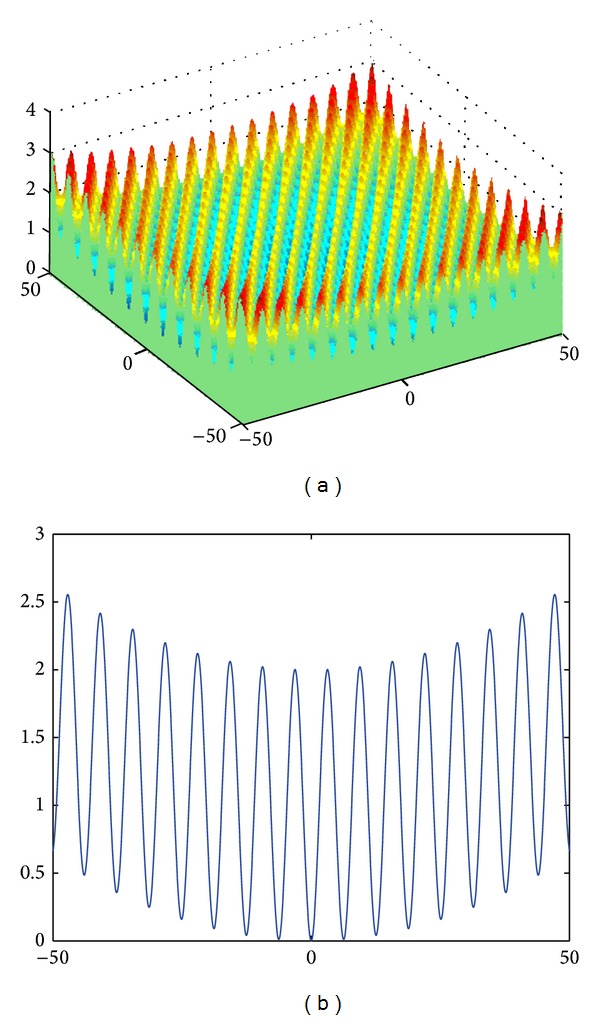
The Griewank function: (a) *n* = 2, (b) *n* = 1.

The formula defining the* n*-dimensional Griewank function (Figure 3) is as follows:

(5) $$\begin{array}{c} f\left(x\right)=\frac{1}{4000}\overset{n}{\underset{i=1}{\sum }}x_{i}^{2}-\overset{n}{\underset{i=1}{\prod }}\cos\left(\frac{x_{i}}{\sqrt{i}}\right)+1. \end{array}$$

**Figure 4:**
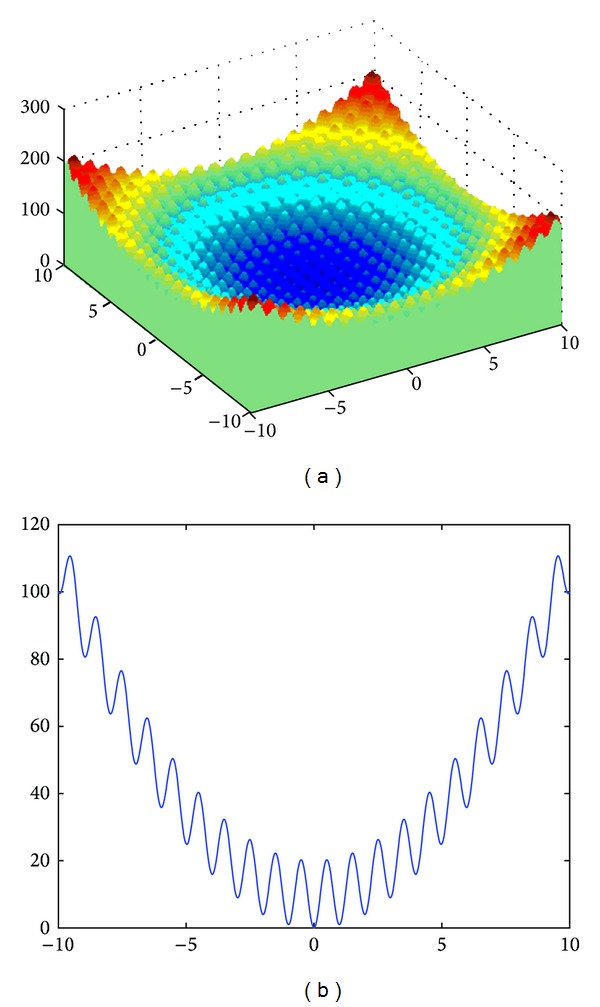
The Rastrigin function: (a) *n* = 2, (b) *n* = 1.

The *n*-dimensional Rastrigin function (Figure 4) is described by the following formula:

(6) $$\begin{array}{c} f\left(x\right)=10n+\overset{n}{\underset{i=1}{\sum }}\left[x_{i}^{2}-10\cos\left(2\pi x_{i}\right)\right]. \end{array}$$

**Figure 5:**
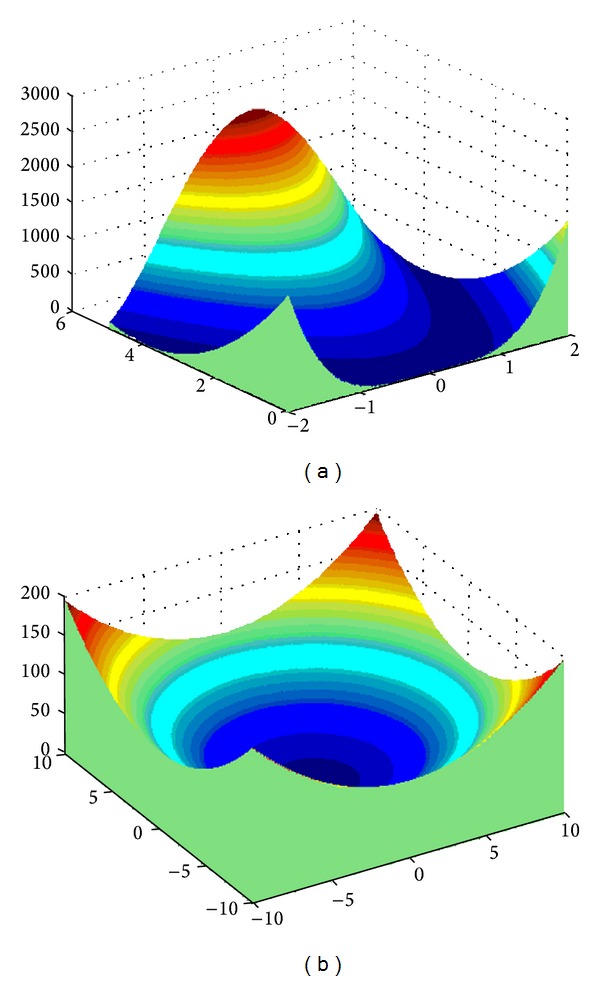
(a) The Rosenbrock function (*n* = 2), (b) the sphere function (*n* = 2).

The following formula defines the *n*-dimensional (*n* > 1) Rosenbrock function (Figure 5(a)):

(7) $$\begin{array}{c} f\left(x\right)=\overset{n-1}{\underset{i=1}{\sum }}\left[100{\left(x_{i+1}-x_{i}^{2}\right)}^{2}+{\left(1-x_{i}\right)}^{2}\right]. \end{array}$$

The sphere function is described by the simple formula (Figure 5(b)):

(8) $$\begin{array}{c} f\left(x\right)=\overset{n}{\underset{i=1}{\sum }}x_{i}^{2}. \end{array}$$

The sphere function is unimodal and multidimensional without local minima, whereas Griewank and Rastrigin functions are multimodal and multidimensional with a huge number of local extremes. The classical Rosenbrock function is a two-dimensional unimodal function, whereas the *n*-dimensional (*n* = 4~30) Rosenbrock function has 2 minima [11]. The global minimum for all functions is equal to 0.

An individual is represented by a vector of a length equal to *n*, where *i*th element contains argument *x*_*i*_ of the optimized *n*-dimensional function (*i* ∈ [1, *n*]).

As was previously explained, in case of minimization problem the formula (3) is used for determining the number of seeds for each individual.

**Figure 6:**
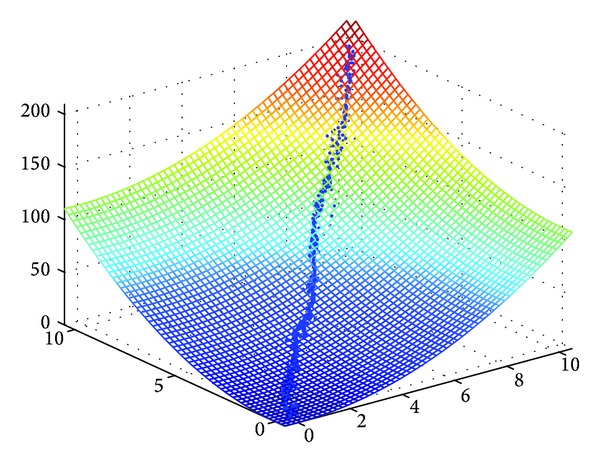
Convergence of exIWO to the optimum of 2-dimensional sphere function.

Following the idea of the IWO authors, the convergence of the exIWO was first tested on the basis of two-dimensional sphere function. Results of the experiment are presented in Figure 6.

**Figure 7:**
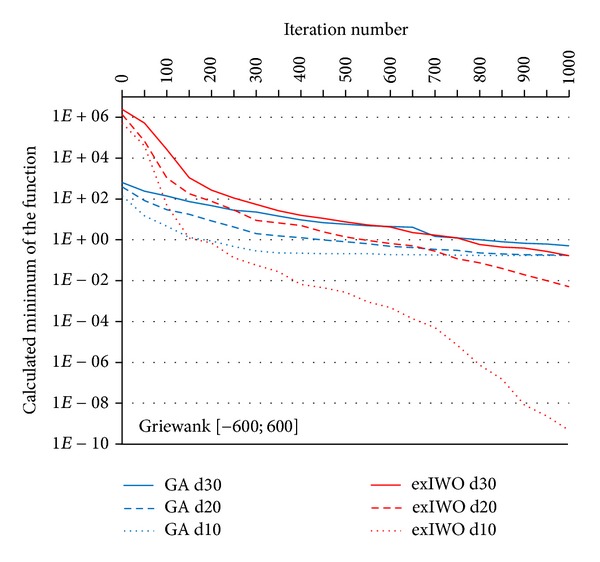
GA and exIWO convergence plot for the Griewank function.

**Figure 8:**
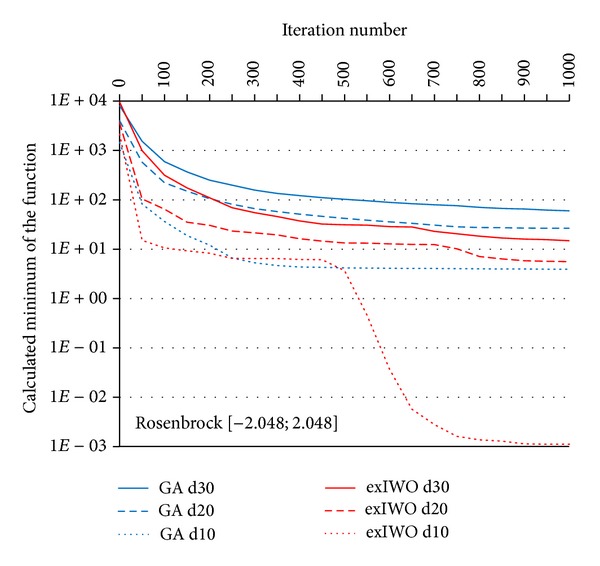
GA and exIWO convergence plot for the Rosenbrock function.

**Figure 9:**
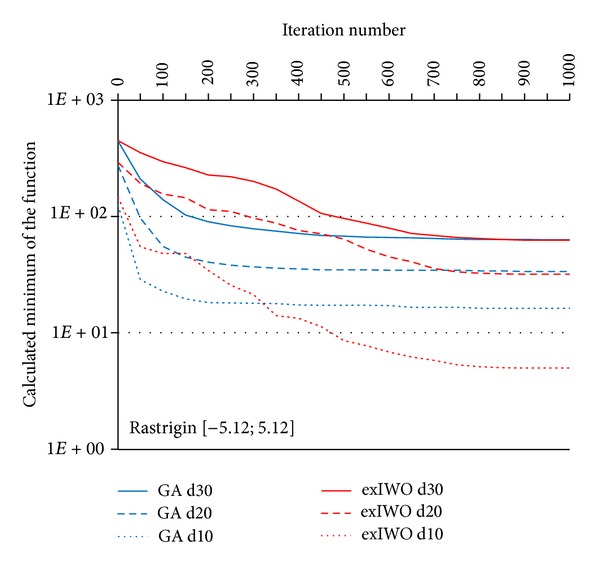
GA and exIWO (global selection) convergence plot for the Rastrigin function.

**Figure 10:**
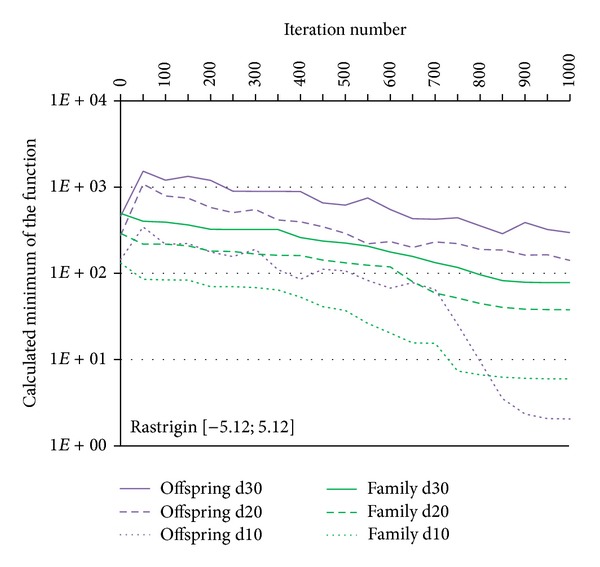
The exIWO convergence plot for the Rastrigin function using offspring-based or family-based selection.

Convergence tests of the exIWO were also performed for *n*-dimensional Griewank, Rastrigin and Rosenbrock functions (*n* = 10, 20, 30). Values of the exIWO parameters were collected in Table 2. They were found during the research as the most appropriate values for the considered functions—the number of trial runs for each function in the presence of a single parameters configuration of the optimization method was equal to 500. However, it is necessary to mention that in the presented convergence tests each population consisted of 20 individuals and the stop criterion was set to 1000 iterations (other values were used in trials which will be discussed later). Analogous tests were carried out with use of the Matlab implementation of GA from the Genetic Algorithm Toolbox [12]. In GA probability of single-point crossover was set to 0.7 and probability of mutation was equal to 0.0017. The results for particular functions along with the initial scope of the search space were shown in Figures 7, 8, 9, and 10 (“*dx*” denotes dimensionality *x*). Consideration should be given to the usage of logarithmic scale.

It should be noted that the selection operator in the GA uses an elitist strategy according to which a predetermined number of individuals with the best fitness values pass to the next generation. This strategy corresponds to the concept of global selection. In case of the exIWO different variants of selection were tested and the global method turned out to be the most promising strategy for all functions except Rosenbrock. However, differences between global and family-based techniques were slight within the scope of the given function. Dissimilarities between selection strategies are shown in Figures 9 and 10 on the basis of the Rastrigin function—the curves representing global and family-based methods descend mildly, whereas offspring-based selection results in nonmonotonic character of the curve caused by the exclusion of the parent individuals from the set of candidates for the next population.

A comparison of GA and exIWO shows that the latter algorithm converges faster in most examined cases.

**Figure 11:**
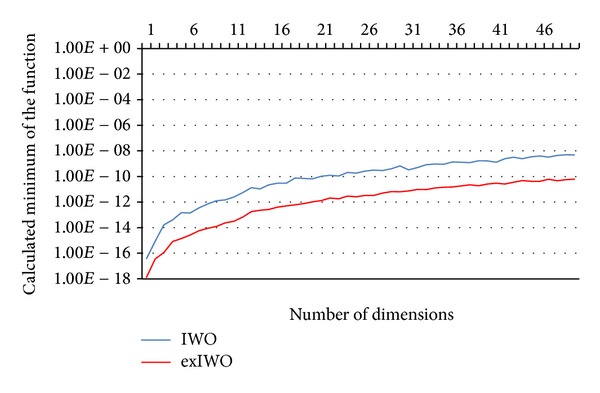
Comparison between IWO and exIWO based on the sphere function.

Experiments related to the numerical functions minimization were also performed for the purpose of comparison of exIWO and IWO. The authors' assumption was to retain conditions proposed for IWO in [3] where only sphere, Griewank, and Rastrigin functions were examined. The parameters of both algorithms used for minimization of the sphere function are included in Table 3. In case of exIWO they were found in a similar manner as described earlier. A single comparative experiment was carried out under the following conditions: A single comparative experiment was carried out under following conditions: stop criterion was determined by the execution time limit equal to 5 [s], dimensionality *n* ∈ [1, 50]; each dimension was limited to [−5.12,5.12]. The number of trial runs for each method was equal to 10. Because of large difference between minima of the sphere function found by both algorithms the logarithmic scale was used in Figure 11.

**Figure 12:**
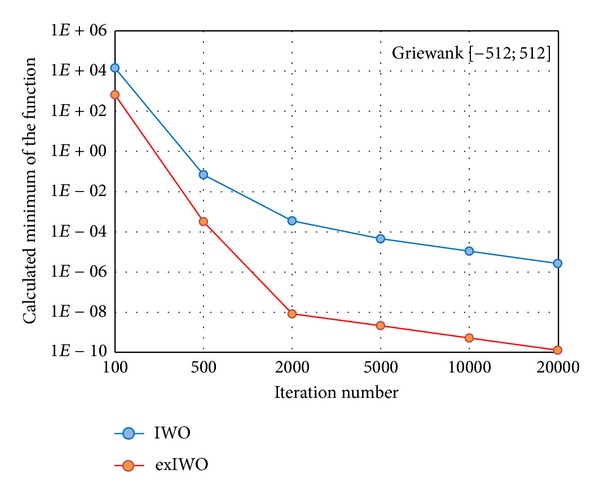
Averaged minima of IWO and exIWO for the 30-dimensional Griewank function.

**Figure 13:**
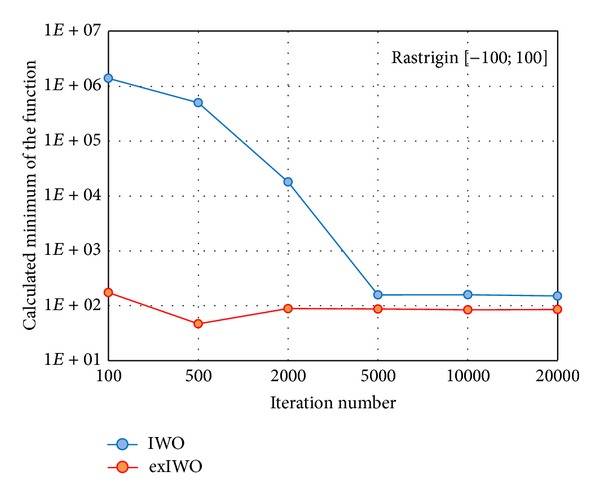
Averaged minima of IWO and exIWO for the 30-dimensional Rastrigin function.

The comparative research on IWO and exIWO was carried on using 30-dimensional Griewank and Rastrigin functions. Similarly to the computations related to the sphere function, the IWO parameters which were collected in Table 4 were taken from [3]. The exIWO parameters are included in Table 2 except the number of iterations which was set in sequence to one of the following values: 100, 500, 2000, 5000, 10000, and 20000 for both algorithms. The minimum values averaged for each experiment's set related to the given number of iterations are presented in the logarithmic scale in Figures 12 and 13.

The exIWO which makes use of the hybrid strategy of the search space exploration obtains better results in comparison to those generated by the IWO.

Results reported in [13] were used for purpose of comparison of exIWO and SPSO. In SPSO candidate solutions are represented by particles forming a swarm. Particles move through the search space and undergo evaluation according to some fitness function. The movements are guided not only by the current locations of particles in the search space, but also by their best locations so far with respect to the fitness function as well as by the best location of the entire swarm. Simple rules for updating position and velocity of each particle allow them to gravitate towards the global extremum [14].

Initial scope of the search space for each argument of particular functions as well as other optimization parameters corresponds with values proposed in [13]. “Asymmetric” character of the initial scope is legitimized by the authors of [15], who state that “Evolutionary optimization algorithms should be tested on benchmark functions in various configurations that include initializing the population with large perturbations directed away from the optimum.”

**Figure 14:**
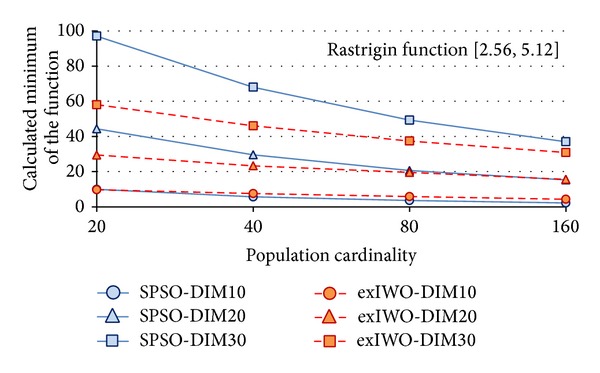
Comparison between SPSO and exIWO based on the Rastrigin function.

**Figure 15:**
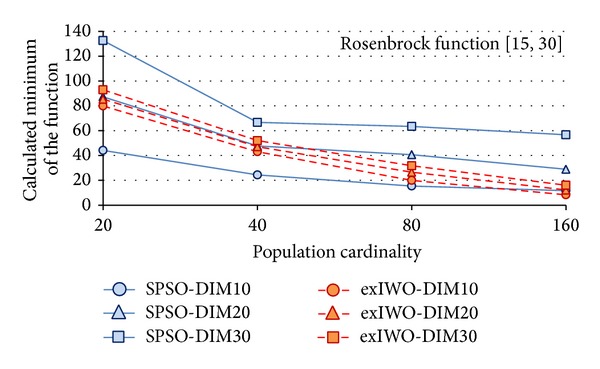
Comparison between SPSO and exIWO based on the Rosenbrock function.

**Figure 16:**
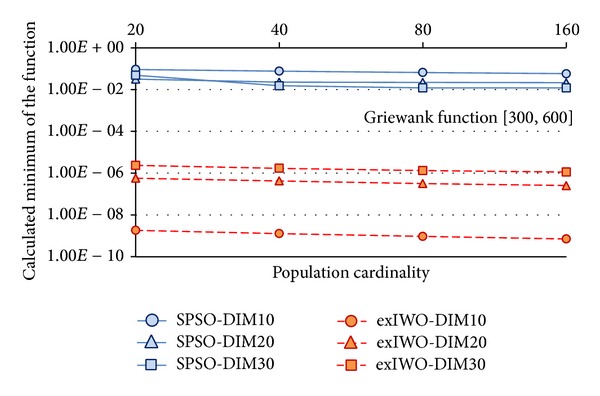
Comparison between SPSO and exIWO based on the Griewank function.

Minima of the *n*-dimensional Rastrigin, Rosenbrock, and Griewank functions (*n* = 10, 20, 30) found by the exIWO algorithm and the SPSO method are presented in Figures 14, 15, and 16, respectively. The number of algorithm iterations (1000, 1500, and 2000) used as a stop criterion is strictly related to the *n* value, as was suggested in [13]. The *x*-axis values denote the number of individuals. Because the minimum of the Griewank function found by the exIWO is several orders of magnitude smaller than the extremum of the same function computed by the SPSO, the logarithmic scale is used for clarity in Figure 16.

The results obtained by the exIWO turned out to be better than the outcomes of the SPSO.

All aforementioned experiments revealed the usefulness of the exIWO for solving continuous optimization problems. The method can compete with other algorithms. Moreover, the hybrid strategy of the search space exploration turned out to be more effective than the method proposed in the IWO.

### 3.2. Feature Selection

According to one of many descriptions of feature (attribute) selection its aim is to choose a subset of features for improving prediction accuracy or decreasing the size of the structure without significantly decreasing prediction accuracy of the classifier built using only the selected features [16]. In other words, attribute selection is expected to simplify object description, discover most discriminative features, and give a chance for more precise classification. Most methods involve searching the space of attributes for the subset that is most likely to predict the class best [17].

The main idea behind the experiments was to test the exIWO ability to find the possibly best subset of features as descriptors of objects subject to recognition: (1) handwritten digits or (2) gait sequences recorded by means of the motion capture technique. Next, the chosen subset was evaluated in terms of accuracy of a supervised classification using only those attributes.

#### 3.2.1. Experiments with Digits

**Figure 17:**
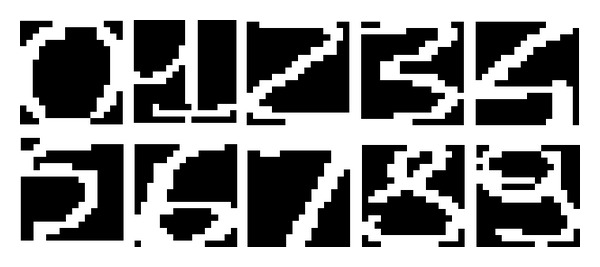
Data sample from the Semeion Handwritten Digit Data Set.

The Semeion Handwritten Digit Data Set (Semeion Research Center of Sciences of Communication, via Sersale 117, 00128 Rome, Italy; Tattile Via Gaetano Donizetti, 1-3-5, 25030 Mairano (Brescia), Italy.), [18] includes 1593 handwritten digits [0–9] which were collected from around 80 persons. Each person wrote all digits twice on paper—trying to write each digit accurately and in a hurry (negligently). Digits were scanned, stretched in a rectangular box 16 × 16 in a gray scale of 256 values and binarized using a simple global threshold equal to 127 (Figure 17). Consequently, each digit is represented by 256 binary features.

#### 3.2.2. Experiments with Gait Sequences

**Figure 18:**
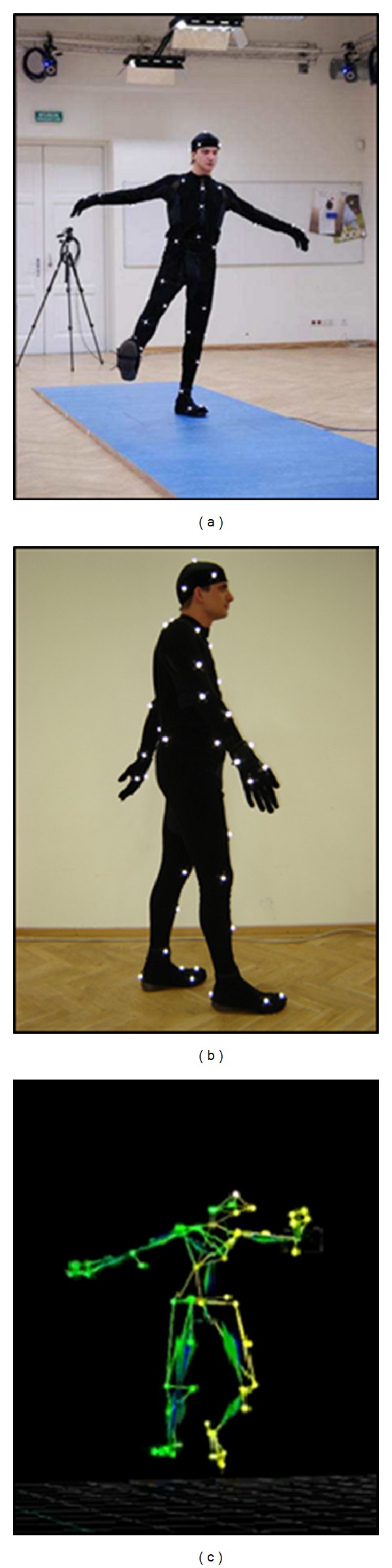
Motion capture recording in the Human Motion Laboratory.

Gait can be captured by two-dimensional video cameras of surveillance systems or by much accurate motion capture (mocap) systems which acquire motion data as a time sequence of poses. In the latter case the movement of an actor wearing a special suit with attached markers is recorded by NIR (Near Infrared) cameras. Positions of the markers in consecutive time instants constitute basis for reconstruction of their 3D coordinates. Gait sequences were recorded in the Human Motion Laboratory (HML) of the Polish-Japanese Institute of Information Technology [19] (Figure 18) by means of the Vicon Motion Kinematics Acquisition and Analysis System equipped with 10 NIR cameras with the acquisition speed of 100 to 2000 frames per second at full frame resolution of 4 megapixels and 8-bit grayscale. The gait route was specified as a 5 meters long straight line. The acquiring process started and ended with a T-letter pose because of requirements of the Vicon calibration process. Two types of motion were distinguished: a slow gait and a fast one. As a result of the acquisition procedure 353 sequences for 25 men aged 20–35 years were stored in a gait database. Motion data lie in high-dimensional space, but the components of gait description are correlated, which allows for dimensionality reduction. Therefore, the mocap data were transformed into the third-order tensor representation required by the Multilinear Principal Component Analysis (MPCA) algorithm [20]. The total number of attributes characterizing a single gait sequence was equal to 8832. After the dimensionality reduction the third-order tensors including 16 features were subject to the feature selection process.

For both problems an individual was represented by a binary vector of a length equal to the initial number of features* n* (*n* = 256 for handwritten digits and *n* = 16 for mocap data).

An individual underwent a transformation which was a simple binary mutation of a randomly chosen element of the vector.

Each weed, that is, each subset of features constructed by the exIWO was used as a set of data descriptors by the 1NN classifier in the supervised classification process. Thus, the fitness function was equivalent to the classification accuracy expressed by means of the Correct Classification Rate (CCR) which indicated the percentage of correctly classified cases. For comparative purposes feature selection was also performed by means of the genetic algorithm as well as the Best-first method—both implemented in the WEKA software [21].

The most appropriate values of the exIWO parameters were collected in Table 5. They were determined in an experimental fashion for each of both the considered problems separately. The number of trial runs for both problems testing a single parameters configuration was limited to 10 because evaluation of a feature selection method based on classification accuracy is rather time consuming. In genetic algorithm probability of crossover was equal to 0.6 and probability of mutation was set to 0.0033. Population cardinality and number of iterations were consistent with values used by the exIWO.

Results of the experiments related to the Semeion Handwritten Digit Data Set are presented in Table 6. The best subset of features selected by the exIWO consisted of 147 attributes and classification accuracy related to its use came to 88.61% (a slightly worse result of 88.23% was obtained by genetic algorithm). The outcomes of the classification based on the entire feature set are as follows [22]: 95.80% (SVM method), 93.35% (boosted C4.5 algorithm), and 76.21% (C4.5).

Results of the experiments on mocap data were collected in Table 7. In case of the mocap gait sequences the best subset selected by exIWO contained 14 features which allowed for gait-based human identification with accuracy of 97.69% independently from the selection method (the same result was obtained by genetic algorithm). Accuracy of the classification based on the entire feature set was lower (CCR = 97.11%) which let supposing that the removed features were useless and caused noise.

Efficiency of the exIWO applied for feature selection does not differ significantly from the results obtained by other tested methods.

### 3.3. Mona Lisa TSP Challenge

**Figure 19:**
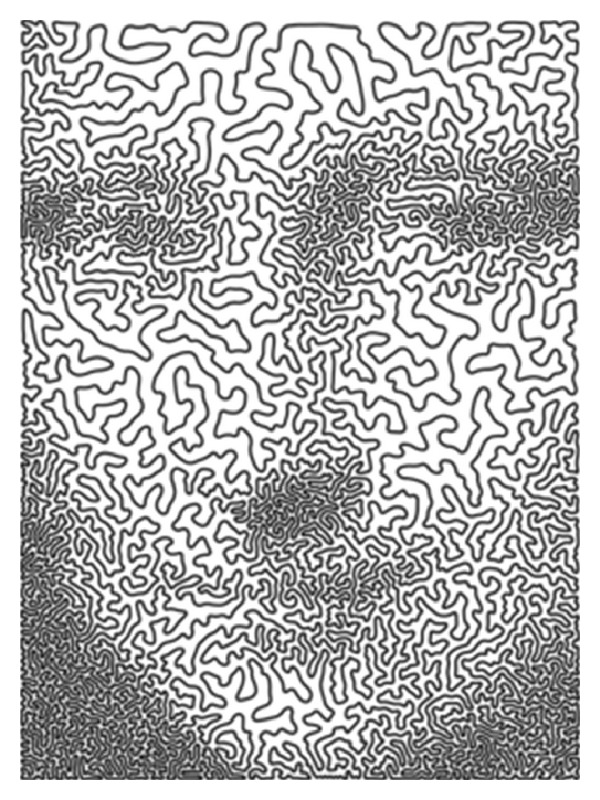
Mona Lisa as a continuous-line drawing [8].

In February 2009, Robert Bosch created a 100000-city instance of the symmetric traveling salesman problem (TSP) that provides a representation of Leonardo da Vinci's Mona Lisa as a continuous-line drawing (Figure 19) [23].

The optimal solution of the TSP is defined as follows [1]: let *C* be a set of *N* cities with distances *d*(*c*_*i*_, *c*_*j*_) ∈ ℝ for each pair of cities *c*_*i*_, *c*_*j*_ ∈ *C*. An optimal solution of the TSP is the shortest tour π* of* C*; that is, a permutation *π* : [1, …, *N*]↦[1, …, *N*] with minimum length *l* = ∑_*i*=1_^*N*−1^ *d*(*c*_*π*(*i*)_, *c*_*π*(*i*+1)_) + *d*(*c*_*π*(*N*)_, *c*_*π*(1)_).

From among significant concepts related to the form of a single solution it is worthwhile to mention three vector representations proposed in the literature:* path*,* ordinal*, and* adjacency* as well as a matrix representation [9]. A plant used by the exIWO was designed according to the simple and natural rule of the path representation: a tour is an ordered list of all cities (i.e., expressed as a vector [239415867]) and the order of visitation is determined by the order of vector elements (2–3–9–4–1–5–8–6–7–2).

The number of seeds for each individual is determined by the formula (3) where length of tour plays the role of cost* K*.

The first population was initialized greedily—for each individual the start city was chosen randomly and the closest city was iteratively added to the tour from among yet unvisited cities.

A single transformation of an individual is based on the* inversion* of a randomly chosen segment of cities. Let *π* be a permutation of *N* cities *π* = (*c*_1_, …, *c*_*p*1_, *c*_*p*1+1_, …, *c*_*p*2−1_, *c*_*p*2_, …, *c*_*N*_), 1 ≤ *p*_1_ ≤ *p*_2_ ≤ *N*. The inversion of the segment between positions *p*_1_ and *p*_2_ leads to the permutation *π*′ such that *π*′ = (*c*_1_, …, *c*_*p*2_, *c*_*p*2−1_, …, *c*_*p*1+1_, *c*_*p*1_, …, *c*_*N*_) [24]. For the exemplary individual [239415867], where the cities 9 and 8 are assumed to be randomly chosen ends of the segment (*p*_1_ = 3, *p*_2_ = 7, hence *c*_*p*1_ = 9, *c*_*p*2_ = 8), the inversion produces a new individual $\left[2\;3\;\underline{8\;5\;1\;4\;9}\;6\;7\right]$ (the inversed fragment was underlined) [9].

The exIWO parameters were selected experimentally.  Table 8 includes the values which resulted in the best Mona Lisa TSP Challenge solution found by the algorithm.

**Figure 20:**
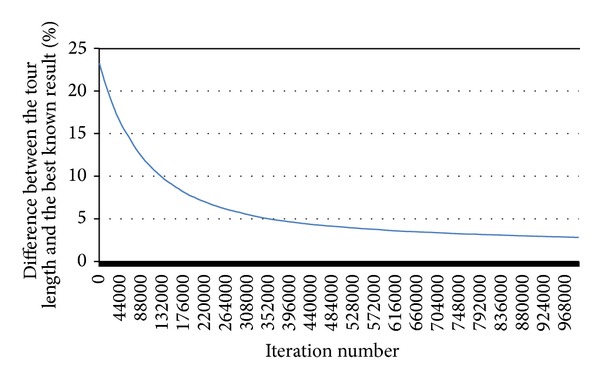
The percentage difference between the length of the current best tour and the best known result for the Mona Lisa TSP Challenge in consecutive iterations.

The tour of length 5 919 404 was found by the exIWO after approximately 19.6 days of computation. It turned out to be worse than the best known result (of length 5 757 191) which was found on March 17, 2009 by Yuichi Nagata [23] and the percentage difference between the lengths of both tours is equal to 2.82% (no tour can have length less than 5 757 084).  Figure 20 illustrates the progressive decrease of the percentage difference between the length of the current best tour and the best known result in consecutive iterations.

It is worthwhile to underline that the final result was achieved by the exIWO making use of greedy method of population initialization and family-based selection in combination with elimination of the spreading from the set of dissemination techniques. This approach was expected to gradually improve “nonaccidental” individuals from the first population.

## 4. Conclusions

The authors of the present paper expanded the idea behind the original IWO algorithm introducing a hybrid strategy of the search space exploration consisting of spreading, dispersing, and rolling down. On one hand the strategy allows for enlargement of the analyzed area of the search space; on the other hand it enables examination of the local extremum in the vicinity of the current point of the space. In addition, two variants of individuals selection were incorporated into the algorithm: (1) the offspring-based technique which should decrease the risk of stagnation at nonoptimal points, (2) the family-based method which should make it possible to preserve features characteristic for a family of individuals.

Results of experiments with both continuous and discrete optimization problems confirmed the versatility of the exIWO; however the adaptation of the metaheuristic for solving the specific problem requires determination of the following components: a representation of a single solution, a method of initialization of the first population, admissible transformations of an individual, a formula of a fitness function, a stop criterion, and a thorough choice of appropriate values of many algorithm parameters.

Because of the time-consuming character of the last operation, future research plans will focus on the adaptive method for tuning of algorithm parameters.

## Figures and Tables

**Algorithm 1:**
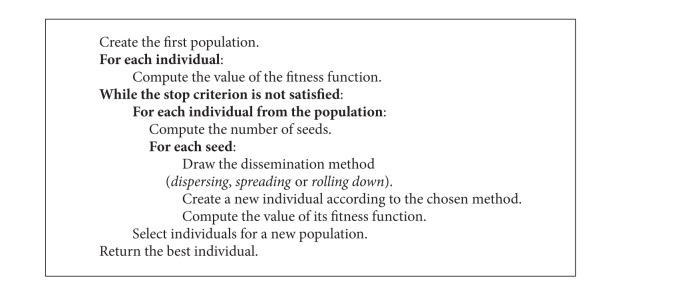


**Algorithm 2:**
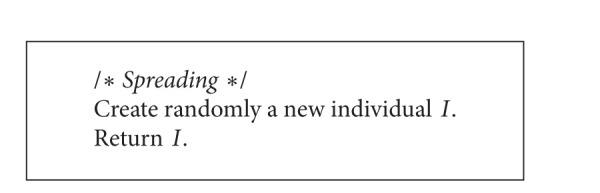


**Algorithm 3:**
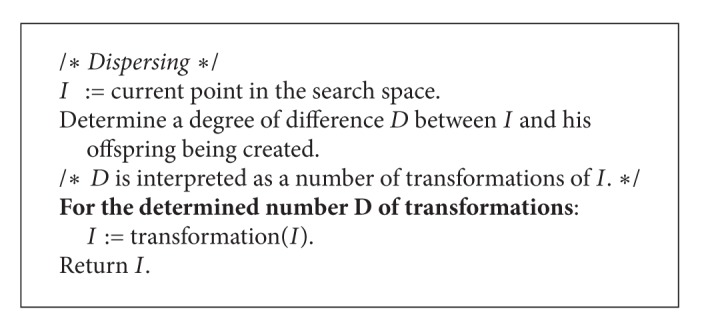


**Algorithm 4:**
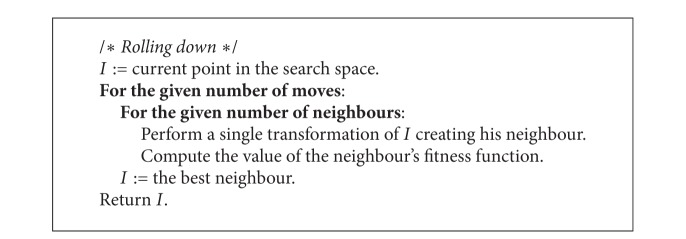


**Table 1:** Main differences between IWO and exIWO.

Component of the algorithm	IWO	exIWO
Initialization of a population	Random	Random or greedy
Spatial dispersal	Dispersing	Spreading, dispersing, and rolling down
Competitive exclusion	Global	Global, offspring-based, or family-based

**Table 2:** The exIWO parameters used for the minimization of numerical functions.

Description	Griewank (*n* = 10)	Griewank (*n* = 20, 30)	Rastrigin	Rosenbrock
Population cardinality	{20, 40, 80, 160}			
Number of iterations (stop criterion)	{1000, 1500, 2000}			
Initialization of the first population	Random			
Max. number of seeds for a weed *S*_max⁡_	5	5	3	6
Min. number of seeds for a weed *S*_min⁡_	0	0	1	0
Initial value of standard deviation *σ*_init_	25	75	25	2.5
Final value of standard deviation *σ*_fin_	0.001	0.005	0.025	0.0075
Nonlinear modulation factor *m*	5	4.5	3	4.75
Number *k* of neighbours and neighbourhoods examined during the rolling down	1	1	1	1
Probability of the dispersing *P*_disp_	0.7	0.3	0.8	0.1
Probability of the spreading *P*_spr_	0.2	0.2	0	0.1
Probability of the rolling down *P*_roll_	0.1	0.5	0.2	0.8
Selection method	Global	Global	Global	Family-based

**Table 3:** The parameters of exIWO and IWO used for minimization of the sphere function.

Description	Value (exIWO)	Value (IWO)
Population cardinality	20	20
Execution time limit (stop criterion) [s]	5	5
Initialization of the first population	Random	Random
Maximum number of seeds sowed by a weed *S*_max⁡_	4	5
Minimum number of seeds sowed by a weed *S*_min⁡_	0	0
Initial value of standard deviation *σ*_init_	0.1	3
Final value of standard deviation *σ*_fin_	0.001	0.001
Nonlinear modulation factor *m*	10	3
Number *k* of neighbours and neighbourhoods examined during the rolling down	1	—
Probability of the dispersing *P*_disp_	0.3	1
Probability of the spreading *P*_spr_	0.3	0
Probability of the rolling down *P*_roll_	0.4	0
Selection method	Global	Global

**Table 4:** The parameters of IWO used for minimization of Griewank and Rastrigin functions.

Description	Value
Population cardinality	20
Number of iterations (stop criterion)	{100, 500, 2000, 5000, 10000, 20000}
Initialization of the first population	Random
Maximum number of seeds sowed by a weed *S*_max⁡_	3
Minimum number of seeds sowed by a weed *S*_min⁡_	0
Initial value of standard deviation *σ*_init_	10
Final value of standard deviation *σ*_fin_	0.02
Nonlinear modulation factor *m*	3
Selection method	Global

**Table 5:** The parameters of exIWO used for feature selection.

Description	Value (digits)	Value (mocap data)
Population cardinality	100	100
Number of iterations (stop criterion)	1000	1000
Initialization of the first population	Random	Random
Maximum number of seeds sowed by a weed *S*_max⁡_	2	2
Minimum number of seeds sowed by a weed *S*_min⁡_	2	2
Initial value of standard deviation *σ*_init_	0.75	8.735
Final value of standard deviation *σ*_fin_	0.01	0.01
Nonlinear modulation factor *m*	6.46	2.59
Number *k* of neighbours and neighbourhoods examined during the rolling down	2	3
Probability of the dispersing *P*_disp_	0	0.4
Probability of the spreading *P*_spr_	0.2	0.2
Probability of the rolling down *P*_roll_	0.8	0.4
Selection method	Offspring-based	Any

**Table 6:** Evaluation of the subset selection mechanisms for the Semeion Handwritten Digit Data Set.

Subset selection mechanism	Classification accuracy (%)	Number of features
exIWO	88.61	147
Genetic algorithm	88.23	138
Best-first	80.89	45

**Table 7:** Evaluation of the subset selection mechanisms for the mocap data.

Subset selection mechanism	Classification accuracy (%)	Number of features
exIWO	97.69	14
Genetic algorithm	97.69	13
Best-first	94.80	8

**Table 8:** The exIWO parameters used for the Mona Lisa TSP Challenge.

Description	Value
Population cardinality	20
Number of iterations (stop criterion)	1000000
Initialization of the first population	Greedy
Maximum number of seeds sowed by a weed *S*_max⁡_	5
Minimum number of seeds sowed by a weed *S*_min⁡_	1
Initial value of standard deviation *σ*_init_	10
Final value of standard deviation *σ*_fin_	0.01
Nonlinear modulation factor *m*	3
Number *k* of neighbours and neighbourhoods examined during the rolling down	3
Probability of the dispersing *P*_disp_	0.8
Probability of the spreading *P*_spr_	0
Probability of the rolling down *P*_roll_	0.2
Selection method	Family-based
